# Intraoperative Identification of Liver Cancer Microfoci Using a Targeted Near-Infrared Fluorescent Probe for Imaging-Guided Surgery

**DOI:** 10.1038/srep21959

**Published:** 2016-02-29

**Authors:** Chaoting Zeng, Wenting Shang, Kun Wang, Chongwei Chi, Xiaohua Jia, Cheng Fang, Du Yang, Jinzuo Ye, Chihua Fang, Jie Tian

**Affiliations:** 1 Department of Hepatobiliary Surgery, Zhujiang Hospital, Southern Medical University, Guangzhou 510280, China; 2 Key Laboratory of Molecular Imaging, Institute of Automation, Chinese Academy of Sciences, Beijing 100190, China; 3 Beijing Key Laboratory of Molecular Imaging, Beijing 100190, China

## Abstract

Difficulties in the highly sensitive detection of tumour microfoci represent a critical obstacle toward improved surgical intervention in liver cancer. Conventional preoperative imaging methods and surgeons’ subjective experience are limited by their inability to effectively detect tumour lesions measuring less than 2 mm; however, intraoperative fluorescence molecular imaging may overcome this limitation. Here, we synthesised an arginine-glycine-aspartic acid (RGD)-conjugated mesoporous silica nanoparticle (MSN) highly loaded with indocyanine green (ICG) dye that could accurately delineate liver cancer margins and provide excellent tumour-to-normal tissue contrast intraoperatively. The increased ICG loading capacity and tumour specificity enabled the identification of residual microtumours and satellite lesions measuring less than 1 mm in living mice. Histological analysis validated the sensitivity and accuracy of this approach. We believe this technique utilising a new fluorescent nanoprobe with intraoperative optical imaging may offer a more sensitive and accurate method for liver cancer resection guidance, resulting in better surgical outcomes.

The outcomes of liver cancer surgeries rely heavily on the accurate localisation of tumour lesions^1^,^2^,^3^. Although imaging modalities such as computed tomography and magnetic resonance imaging offer valuable preoperative diagnostic information, effective detection of tumour microfoci (<2 mm) of satellite tumour nodules or postoperational residues remains challenging due to limitations in imaging sensitivity^4^,^5^,^6^. Recent advances in near-infrared (NIR) fluorescence molecular imaging (FMI) for intraoperative image-guided cancer resection have introduced new frontiers for FMI-based therapeutic interventions in preclinical research^7^,^8^,^9^,^10^ and clinical applications^11^,^12^,^13^. The inherent advantages of this novel technology, such as its high sensitivity, high superficial resolution, low cost, and real-time imaging capacity, have stimulated the development of fluorescent probes with different molecular features^4^,^14^,^15^. These probes must offer sufficient optical contrast between the lesion and its surrounding normal tissue, normally quantified as the tumour-to-background ratio (TBR), to adequately distinguish tumour microfoci.

One strategy for obtaining better TBR in hepatic tumour sites has been to increase the tumour specificity of the fluorescent probe^16^,^17^,^18^,^19^, allowing a higher proportion of the intravenously (IV) administered probe to accumulate in the tumour. The other strategy applies fluorescent substances (organic fluorophores or inorganic nanoparticles) with better optical properties (e.g., higher quantum yield and/or better emission spectrum) to synthesise fluorescent probes^8^,^20^,^21^,^22^ with enhanced detectability. Unfortunately, since the relatively small volume of a microtumour limits its uptake of a molecular probe due to the lower overall expression of the target antigen, it is challenging to achieve effective detection of tiny tumour foci in large organs using these conventional strategies during hepatic surgery. Thus, novel designs and/or optimised preparation methods are necessary for effective clinical application.

Mesoporous silica nanoparticles (MSNs) have received significant attention as drug nanocarriers in cancer therapy due to their unique properties, including high chemical stability, controllable pore size, large surface area, and superb drug loading ability^23^,^24^. Furthermore, silica is generally viewed as safe for use in biomedical applications by the United States Food and Drug Administration (FDA)^25^, and its biocompatibility has been demonstrated^24^,^26^,^27^. However, the application of MSN-based nanoparticles in NIR-FMI-guided liver cancer surgery remains underexplored. Consequently, we hypothesised that MSNs, as nanocarriers conjugated with multiple NIR fluorophores, could serve as novel probes to augment the overall quantum efficiency upon delivery to the targeted site. This strategy could provide high optical contrast for tumour microfoci with limited receptor-molecule expression.

In this study, we synthesised arginine-glycine-aspartic acid (RGD)-conjugated highly loaded indocyanine green (ICG) MSNs (ICG/MSNs-RGD) as a fluorescent probe and explored its application to intraoperative NIR-image-guided liver tumour resection. RGD is a peptide ligand for the integrin α_v_β_3_ receptor that is overexpressed in many tumour types^28^,^29^. Through systematic evaluation, our probe accurately delineated tumour margins and detected microtumours due to its excellent optical contrast properties. The average size of the detected microfoci was 0.4 ± 0.21 mm. Our results established that ICG/MSNs-RGD and the underlying strategy could provide surgeons with objective guidance for the precise removal of microfoci during hepatic surgery.

## Results

### Synthesis and characterisation of ICG/MSNs-RGD

**Figure 1:**
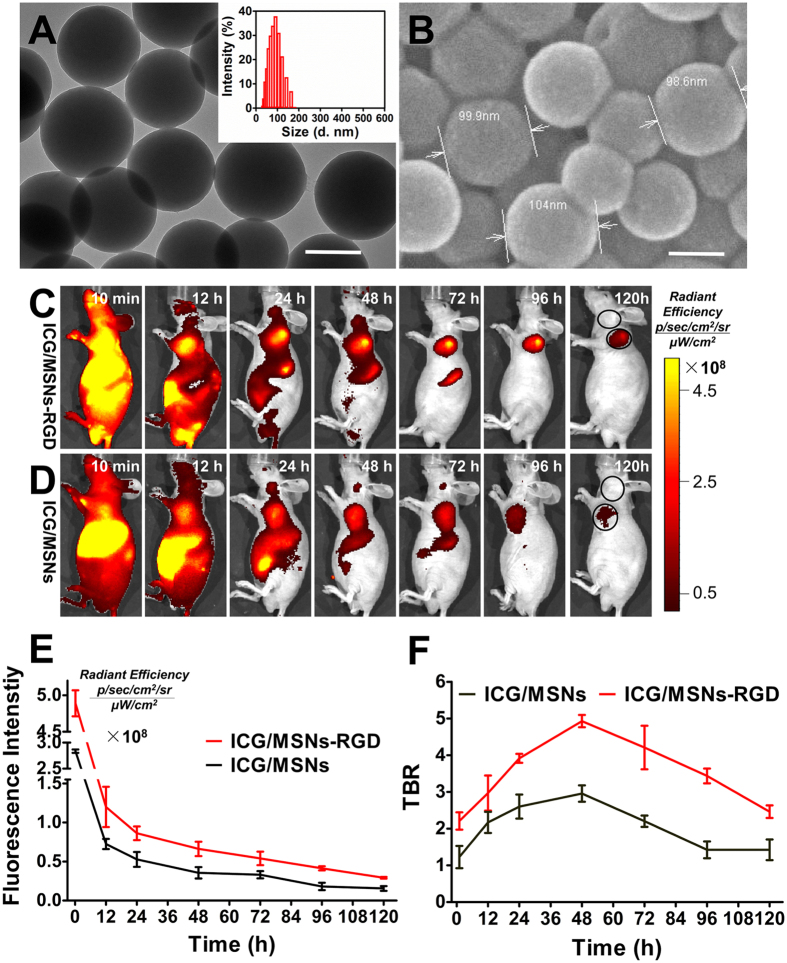
Characteristics of MSNs and ICG/MSNs-RGD and RGD-mediated tumour specificity *in vivo*. (**A**) TEM image of MSNs showing a typical spherical structure and average particle size. The inset shows the particle size distribution of ICG/MSNs-RGD. Scale bar, 100 nm. (**B**) SEM image of MSNs confirmed the TEM findings in (**A**). *In vivo* continuous observations (120 h) of liver cancer xenografts after administration of ICG/MSNs-RGD (**C**) and ICG/MSNs (**D**) using FMI. Black circles indicate the regions of interest for calculating the TBR at each time point. (**E**) Quantification of the fluorescence intensity at the tumour sites showing accumulation of ICG/MSNs-RGD. (**F**) Comparison of TBR profiles of the two probes. Experiments were run in triplicate.

The size and morphology of a nanoprobe are critical factors affecting tumour targeting *in vivo*, and particles with sizes between 30 and 150 nm are particularly favorable^30^. The shapes of the MSNs and ICG/MSNs-RGD were characterised using transmission electron microscopy (TEM) and scanning electron microscopy (SEM). The results revealed that the MSNs and ICG/MSNs-RGD exhibited a typical spherical morphology with an average diameter of less than 100 nm (Fig. 1A,B). The average hydrodynamic diameter of the ICG/MSNs-RGD was approximately 100 nm, as measured by DLS (Fig. 1A).

Similar to ICG (Fig. S2A), the excitation spectrum of ICG/MSNs-RGD exhibited an absorption peak at 770 nm and a fluorescence emission at 837 nm. The ICG/MSNs-RGD exhibited excellent stability in foetal bovine serum (FBS; Fig. S2B). *In vitro* cytotoxicity testing of the nanoparticles indicated that it has good biocompatibility (Fig. S3A). The data for the cellular uptake of the RGD-based probes indicated that the ICG/MSNs-RGD probes could combine with the integrin α_v_β_3_ receptor specifically and that the ICG/MSNs-RGD probes had satisfactory tumour specificity (Figs S3B, C and S4). More details are available in the Supplemental Data.

### *In vivo* validation of RGD-mediated tumour specificity

The tumour specificity of the ICG/MSNs-RGD *in vivo* was validated in mice bearing subcutaneous Hep-G2-green fluorescent protein (GFP)-fLuc tumours with high integrin α_v_β_3_ expression. Two groups of three mice were injected intravenously (i.v.) with either the ICG/MSNs or ICG/MSNs-RGD probe (150 μL, 0.2 mg/mL). Continuous fluorescence imaging over 120 h indicated marked accumulation of both probes in tumour lesions up to 96 h; however, the ICG/MSNs-RGD probe displayed better optical contrast than the ICG/MSNs in the tumour region from the 24 h time point onward (Fig. 1C,D). After the initial distribution period (<12 h), the majority of the optical signal was emitted from the tumour and abdominal areas; the signal then gradually faded after 24 h postinjection. These results suggested that the probes were excreted through the liver and digestive system, in accordance with our expectations for a probe this size (100 nm)^30^.

Quantification of the radiant efficiency showed that ICG/MSNs-RGD displayed a significantly stronger optical signal in tumour lesions at all observation points (*p* < 0.05, Fig. 1E), verifying its excellent *in vivo* targeting ability for integrin α_v_β_3_-overexpressing tumours. In addition, significantly different TBR profiles were observed between the two probes (Fig. 1F). Notably, the ICG/MSNs-RGD TBR peak (4.92 ± 0.2) appeared at 48 h after injection and was ~1.7-fold higher than that of ICG/MSNs (2.9 ± 0.2) at the corresponding time. An *ex vivo* comparison of the biodistribution between the targeted and untargeted fluorescent probes was performed on major organs 48 h after i.v. injection, which again demonstrated the higher tumour uptake of ICG/MSNs-RGD (Fig. S6). These data confirmed the tumour specificity of ICG/MSNs-RGD and indicated that surgeries should be performed 48 h after probe injection for optimal NIR-FMI guidance.

### Intraoperative tumour margin definition

**Figure 2:**
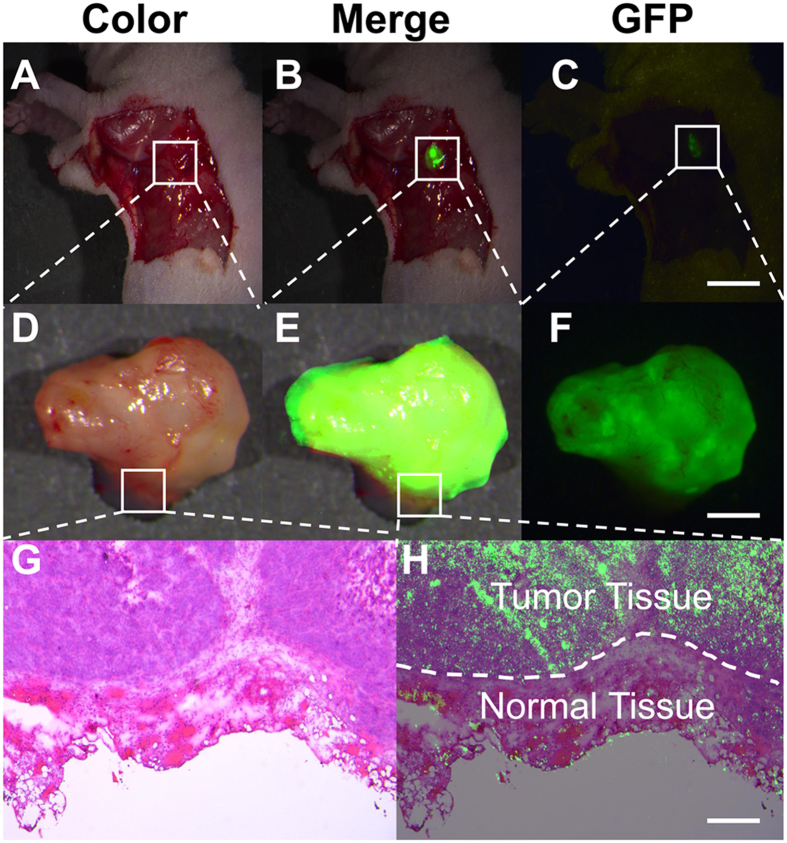
Intraoperative tumour margin definition. *In vivo* and *ex vivo* tumour margin definition using ICG/MSNs-RGD. (**A**) The white-light image of the exposed tumour lesion did not provide clear tumour-to-normal tissue contrast. (**B**) ICG/MSNs-RGD provided distinct optical contrast at the tumour margin with intraoperative NIR-FMI. (**C**) The reference GFP image also confirmed the findings of the ICG/MSNs-RGD in (**B**). Scale bar, 8 mm. (**D**) With NIR-FMI guidance, the tumour tissue was removed by a surgeon. (**E,F**) Both NIR and GFP images provided consistent tumour margin definition of the resected tissue. Scale bar, 2 mm. (**G**) Histological analysis of the tumour specimen encompassed the border regions between normal and tumour tissues from the resected tissue in (**D**). (**H**) Overlay of the NIR-fluorescent image and H&E staining of the specimen, demonstrating consistent tumour margin definition at the microscopic level. Scale bar, 100 μm. Experiments were run in triplicate.

The feasibility of ICG/MSNs-RGD-mediated intraoperative NIR image guidance was first investigated in mice bearing subcutaneous hepatocellular carcinoma (Hep-G2-GFP-fLuc) xenografts. The tumour margins were very difficult to visually identify after removing the skin (Fig. 2A); however, the ICG/MSNs-RGD provided remarkable optical contrast at the tumour margin by NIR-FMI (Fig. 2B), which was confirmed by both NIR (840 nm) and GFP (500 nm) imaging *in vivo* (Fig. 2B,C), as well as with the excised tumours (Fig. 2D–F). To further investigate the accuracy of the tumour contrast with ICG/MSNs-RGD, histological analyses were performed on resected tissues encompassing the border regions between normal and tumour tissues (Fig. 2G,H). The tumour margin definitions given by both NIR illumination and hematoxylin and eosin (H&E) staining were perfectly overlapped at the microscopic level (Fig. 2H).

Quantitatively, NIR imaging showed a notable improvement in the TBR over that of GFP (6.0 ± 0.8 versus 4.0 ± 0.3, respectively; Fig. 2B,C). Considering the difference in exposure times (2 versus 30 s, respectively), the optical contrast was much greater than it appeared in these images. Collectively, these data demonstrated the advantage of the high ICG loading capacity of the MSN nanocarriers.

### Intraoperative tumour residual detection

**Figure 3:**
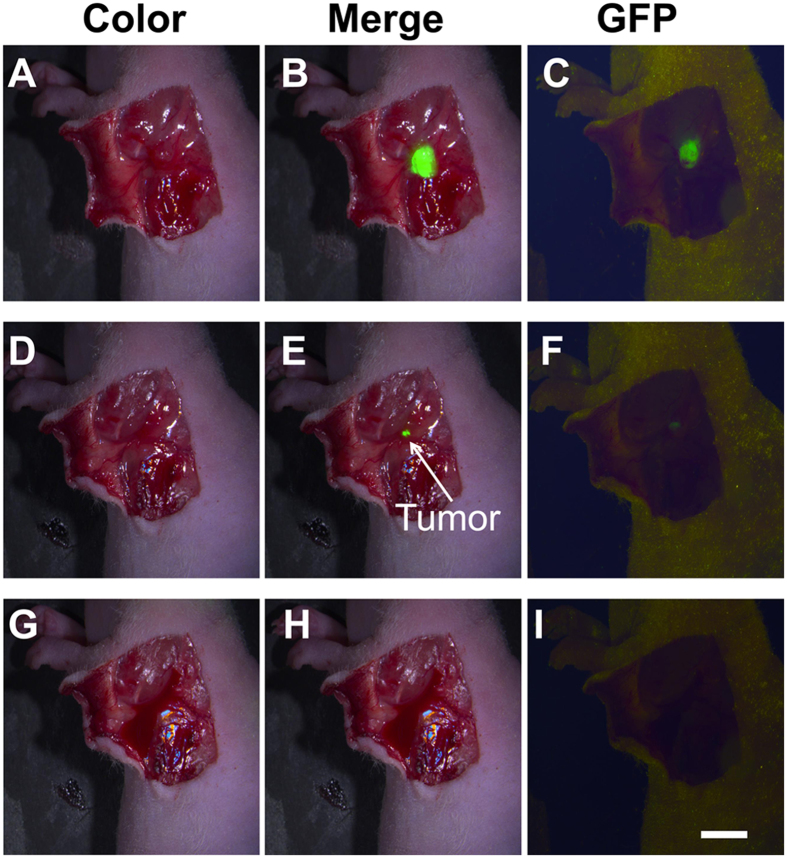
Intraoperative detection of residual tumour tissue. An example of surgical tumour residual assessment. (**A–C**) Both NIR and GFP fluorescent images confirmed the tumour lesion before the resection. (**D**) The surgeon determined that the tumour tissue was removed without the help of intraoperative NIR-FMI. (**E,F**) Both NIR and GFP confirmed there was a microtumour residual, which was completely unrecognisable in (**D**). Notably, the NIR image offered better tumour-to-normal tissue contrast after a shorter exposure time (NIR versus GFP: 2 versus 40 s, respectively). (**G–I)** With ICG/MSNs-RGD-mediated NIR-FMI, the surgeon removed the micro residual completely (**H**), which was also confirmed by the GFP image in (**I**). Experiments were run six times for each surgeon (two surgeons participated). Scale bar, 7 mm.

Because ICG/MSNs-RGD offered excellent optical contrast and accurate tumour margin definition, we next investigated its potential for intraoperative imaging. Two experienced surgeons were assigned twelve Hep-G2-GFP-fLuc xenografts (six mice per surgeon) to completely remove the tumour lesion while minimising surgical trauma. NIR and GFP fluorescence images were obtained before and after the resection to verify tumour residuals (Fig. 3). However, the tumour residuals information was not given to the surgeons until they determined whether the tumour was removed completely. For Surgeons 1 and 2, three and two residual microtumours were detected, respectively. While it was not difficult to remove major tumour tissues, i.e., 5.01 ± 2.08 mm in size (Fig. 3A–C), it was impossible for the surgeons to identify residual microfoci, i.e., those measuring less than 0.95 ± 0.07 mm in size (Fig. 3D–F). However, the use of ICG/MSNs-RGD-mediated intraoperative NIR-FMI provided sufficient optical contrast and highly sensitive detection of small lesions, allowing for the full removal of tumour tissues (Fig. 3G–I). Pathological examination of the resected tissues confirmed that all specimens (n = 17) were true positive tumours.

### Intraoperative detection of satellite tumour nodules

**Figure 4:**
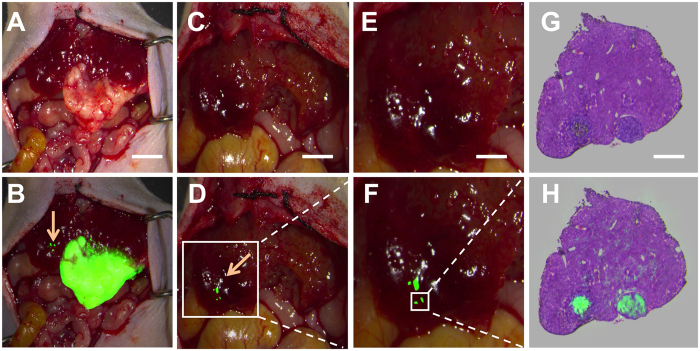
Intraoperative detection of satellite tumour nodules. Highly sensitive detection of satellite tumour nodules. (**A**) The implanted liver tumour tissue exhibited obvious contrast in colour and texture with normal liver tissues. Scale bar, 3 mm. (**B**) ICG/MSNs-RGD-mediated NIR-FMI confirmed the position and margin of the tumour tissue and showed the presence of several microtumour foci (yellow arrow) separate from the major tumour tissue with remarkable optical contrast. (**C**) The major tumour tissue was resected. Scale bar, 2 mm. (**D**) The microtumour foci that were not noticeable in (**C**) were identified again by NIR-FMI. (**E,F**) With proper magnification, these satellite foci were even more clearly and sharply defined. Scale bar, 1 mm. (**G,H**) H&E staining and NIR fluorescence imaging of the specimen indicated the microtumour uptake of ICG/MSNs-RGD. Scale bar, 500 μm. Experiments were run in triplicate.

Having established the efficacy of intraoperative NIR-FMI with ICG/MSNs-RGD, we sought to investigate the performance of this approach for the detection of liver cancer metastases and satellite foci. Intraoperative NIR-FMI-guided surgery was performed on three mice with intrahepatic metastases 48 h after the i.v. administration of ICG/MSNs-RGD (0.2 mg/mL, 150 μL). Notably, only the implanted tumours (5.09 ± 2.31 mm) could be visually recognised based on the surgeons’ subjective experience after exposing the liver (Fig. 4A), and NIR imaging confirmed this with accurate tumour margin definition (Fig. 4B). Furthermore, NIR-FMI enabled the accurate detection of microtumour lesions (0.4 ± 0.21 mm) with excellent optical contrast (TBR: 4.7 ± 0.21; Fig. 4B,D,F). These highly suspicious satellite foci were impossible to identify in the absence of ICG/MSNs-RGD (Fig. 4A,C,E). Moreover, H&E staining and NIR fluorescence imaging of the specimens confirmed all foci as tumour nodules, and ICG/MSNs-RGD was indeed accumulated in the tumour lesions (Fig. 4G,H).

**Figure 5:**
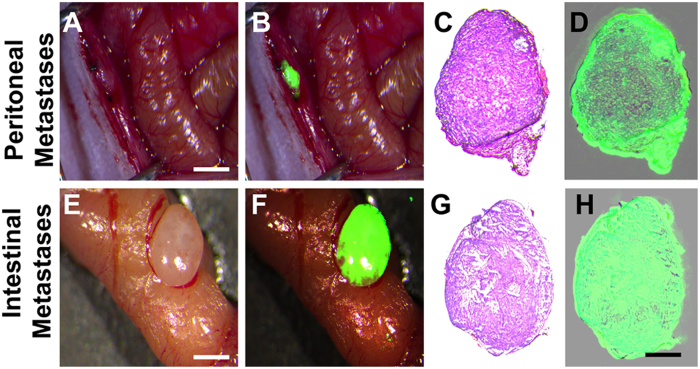
Intraoperative detection of satellite tumour nodules. Unexpected detection of peritoneal satellite lesions. (**A,B**) Intraoperative detection of a satellite lesions in the peritoneum. Scale bar, 1 mm. (**C,D**) Histological and fluorescence analysis of the resected tissue specimen from (**B**). Scale bar, 500 μm. (**E,F**) Intraoperative detection of a satellite lesion on the intestinal wall. Scale bar, 1 mm. (**G,H**) Histological and fluorescence analysis of the resected tissue specimen from (**F**). Scale bar, 500 μm. Experiments were run five times.

Unexpectedly, we found five other mice with peritoneal satellite lesions, likely due to the leakage of tumour cells during implantation. The average size of these lesions in the peritoneum was 1.32 ± 0.27 mm (Fig. 5A,B), whereas others on the intestinal wall were 0.91 ± 0.13 mm in size (Fig. 5E,F). The TBR of all satellite lesions was 5.23 ± 0.82. Significantly, these sites were easily identified by the surgeon during the NIR image-guided surgeries and were later confirmed to be true positive tumour tissues with subsequent histological and fluorescence analysis (Fig. 5C,D,G,H).

As the first capillary bed to be encountered by circulating malignant cells, the liver commonly contains metastases from primary tumours in other organs. Therefore, we also simulated liver metastasis from breast cancer, which is the second most common hepatic metastasis in clinical practice^31^,^32^,^33^, by transplanting human breast cancer cells (MDA-MB-231-fLuc) into mouse livers (n = 4). After the administration of ICG/MSNs-RGD, the intraoperative NIR-FMI successfully detected the transplanted tumour lesions in all mice (Fig. S7A,B). All the detected tumour lesions were histologically confirmed (Fig. S7C). These data indicated the great potential of using ICG/MSNs-RGD intraoperatively for a wide range of clinical scenarios during liver cancer surgery.

## Discussion

The effectiveness of intraoperative fluorescence imaging guidance has been validated in various preclinical and clinical applications^34^,^35^,^36^. Clinically, image-guided surgery based on ICG has been applied to map sentinel lymph nodes of tumours arising in the breast, skin, gastrointestinal tract, lung, and other organs^37^,^38^,^39^,^40^. Furthermore, the ICG has been used for intraoperative imaging of hepatocellular tumours^12^. The technique provides valuable information, crucial for improving surgical outcomes. Nevertheless, a fluorescent probe capable of detecting tumour microfoci (<2 mm) is required to further the clinical applications of this modality. Here, we describe the design and potential of ICG/MSNs-RGD, which can be used intraoperatively for NIR-FMI-guided surgery, to accurately detect and precisely remove easily missed residual microfoci or tiny tumour metastases.

The RGD-modified probe targeted integrin α_v_β_3_-overexpressed tumour cells, and its specificity was verified both *in vitro* and *in vivo*. With the specific binding of the RGD peptide to integrin α_v_β_3_, the nanomaterials could be internalised into cells by clathrin-mediated endocytosis, also known as receptor-mediated endocytosis (RME). Several studies have reported that RGD-containing nanomaterials could enhance cellular uptake through the RME pathway^41^,^42^. RGD, when combined with the peptide ligand for neuropilin-1 (NRP1), a transmembrane receptor, was found to promote selective tumour vascular targeting and facilitate the penetration of drugs and imaging agents into the tumour^17^,^29^,^43^. Accordingly, ICG/MSNs-RGD provided accurate intraoperative tumour margin definition, as corroborated by histological analyses. The probe was synthesised from biocompatible materials (MSNs and ICG) and is thus inherently safe. Indeed, cytotoxicity assays confirmed its biocompatibility (Fig. S3), and 120 h of continuous observation after *in vivo* administration demonstrated its biodistribution, clearance, and elimination.

In addition to these demonstrated advantages, ICG/MSNs-RGD was specifically designed to offer sufficient optical contrast for tumour microfoci, despite the limitations of probe uptake by small tumour lesions. This was achieved by employing MSNs as nanocarriers that encapsulated the ICG; for each successfully delivered ICG/MSNs-RGD nanoparticle, multiple ICG molecules could be effectively activated and contribute to the optical contrast between tumour and normal tissues. After adjusting the MSN mesoporosity to 5 nm, the encapsulation efficiency reached an extraordinary 37.5% (Fig. S2C). The highest expected MSN loading efficiency was ∼18.2–32%^24^,^44^, and an efficiency of 32% should have produced MSNs exceeding 100 nm^24^,^45^. Compared with these studies, we enhanced the loading capacity while reducing the size of the nanoprobe. This high ICG loading ability ensured that ICG/MSNs-RGD could pinpoint tiny tumour foci intraoperatively.

To prove our hypothesis, we established different liver cancer-bearing mouse models and applied intraoperative NIR-FMI with ICG/MSNs-RGD to simulate various clinical scenarios. The experiments conducted by the two surgeons demonstrated that the probe facilitated effective detection of liver cancer residuals less than 1 mm in size. The tumour metastasis experiment also indicated that a surgeon can easily recognise intrahepatic and extrahepatic liver cancer satellites of less than 1 mm using the newly developed probe. This is likely the most sensitive intraoperative NIR-FMI performance in liver cancer surgery to date. Moreover, ICG/MSNs-RGD provided TBR values for the liver microtumours that exceeded 4.5, which revealed the superb optical contrast between the tumours and surrounding tissues. These experiments proved that ICG/MSNs-RGD could provide extremely high sensitivity for the intraoperative detection of tumour foci at the micrometre level, while maintaining a field of view encompassing more than half of the body of the mouse.

As in other *in vivo* NIR imaging studies, the optical signal penetration depth is the major limitation of this technique, which constrains effective liver tumour detection to superficial lesions^21^,^34^. The current NIR-FMI system has been used for imaging separate from a standard colour imaging system, and surgeons would be expected to switch between bright-field and fluorescence modes in the operating room in order to use the NIR fluorescent probes. However, with advances in technology, fluorescence imaging systems and colour imaging systems can be used simultaneously in real time. In additionally, the ICG/MSNs-RGD may not be applicable to all types of cancers, except tumours exhibiting overexpression of integrin α_v_β_3_. However, dual-targeted nanoparticles may be a powerful tool for overcoming these problems. A recent report described a dual-targeted probe (cyclic-RGD-PLGC(Me)AG-ACPP) that could be taken up by multiple types of human cancer cells and delivered more therapeutic or diagnostic agents to tumour regions^46^.

ICG/MSNs-RGD can greatly assist surgeons in the intraoperative detection of liver cancer. Accurate delineation of tumour borders and sensitive detection of tumour residuals are both benefits of the high ICG loading capacity of the MSNs. This molecular design strategy can be extended to other tumour imaging applications. Through the conjugation of different targeting domains (peptides or antibodies), this method has great potential in the highly sensitive and accurate visualisation of tumour microfoci with expression of various biomarkers^3^,^47^. Thus, the ability to pinpoint microtumour foci with superb optical contrast at the macroscopic level is valuable for clinical applications and may provide promising assistance for intraoperative diagnostic and therapeutic interventions.

In this study, we successfully constructed an active tumour-selective probe, ICG/MSNs-RGD, using MSNs with a high ICG-loading ability and high α_v_β_3_ receptor targeting specificity. The probe demonstrated precise tumour margin delineation and highly sensitive microfoci detection during liver cancer surgery. Both tumour metastases and surgical residuals at the micrometre level were effectively captured intraoperatively with a macroscopic field of view. Furthermore, the probe exhibited promising biocompatibility, good optical stability, and high water dispersibility. We believe this novel fluorescent nanoprobe with the new high-fluorochrome-loading design strategy will afford more sensitive and accurate guidance of liver cancer resection to achieve better surgical outcomes.

## Materials and Methods

### Synthesis and characterisation of ICG/MSNs-RGD

MSNs with customisable pore and particle sizes were generated by hydrothermal synthesis^24^,^26^. Briefly, cetyltrimethylammonium bromide (250 mg, CTAB, 98%; Alfa Aesar, China) was dissolved in ultrapure water (120 mL), and NaOH (0.5 M, 875 μL) was added. After adjusting the temperature to 80 °C and stirring for 5 min, tetraethoxysilane (300 μL, TEOS, 98%; Alfa Aesar) was added dropwise with constant stirring. The reaction proceeded for 20 min. Then, 3-aminopropyltriethoxysilane (100 μL, APTES, 99%; Acros, USA) was added, and the system was maintained at 80 °C for 2 h. The reaction supernatant was centrifuged at 12000 rpm for 25 min. The resulting NH_2_-SiO_2_ was collected, extensively washed with ultrapure water and absolute ethyl alcohol, and then dried under high vacuum at 30 °C overnight to remove any remaining solvent.

The ICG/MSNs-RGD probes were synthesised by adding NH_2_-SiO_2_ (10 mg) to ICG solution (1 mL, 10 mg/mL; Dandong Medical and Pharmaceutical Co. Ltd., Dandong, China) and vibrating overnight (Fig. S1). After the suspension was centrifuged at 12000 rpm for 25 min, the supernatant was used to calculate the loading efficiency by ultraviolet-visible (UV-Vis) spectroscopy (UV-2450; Shimadzu, Japan), using the following formula: loading efficiency (%) = (drug input [mg] – drug in supernatant [mg])/drug input (mg) × 100. ICG-loaded MSNs (ICG/MSNs) were resuspended and vibrated in polyethylene glycol (5 mL, 5000 MW, 1 mg/mL; Alfa Aesar) overnight. The RGD peptide (Arg-Gly-Asp, 5 mg, 98%; Beijing, China) was dissolved in the ICG/MSNs solution (1 mL) and vibrated for 6 h. The ICG/MSNs-RDG product vial was stored at room temperature, protected from light.

TEM (JEOL-1011; JEOL, Japan) and SEM (HITACHI S-4800; Hitachi, Japan) were used to evaluate the morphology and size of the MSNs and ICG/MSNs-RGD, with a 100 kV acceleration voltage. The hydrodynamic particle size distribution was measured by dynamic light scattering (DLS) using a Malvern Zetasizer (ZEN 3600; Malvern, UK). The fluorescence excitation and emission spectra of the ICG and ICG/MSNs-RGD were measured with a fluorescence spectrofluorometer (F-7000; Hitachi). For stability analysis, the ICG/MSNs-RGD (50 μg) was added to FBS (1 mL; Gibco, Australia) and its optical absorbance at 780 nm was monitored by UV-Vis spectroscopy (UV-2450; Shimadzu) at multiple time points for 24 h. A serum-only control solution was also monitored.

### Cell cytotoxicity assays

The cellular cytotoxicity of the MSNs and ICG/MSNs-RGD was assessed by 3-(4,5-dimethylthiazol-2-yl)-2,5-diphenyltetrazolium bromide (MTT) assay (Promega, China). GFP-transfected luciferase-expressing human hepatocellular carcinoma cells (Hep-G2-GFP-fLuc cells), which expressed αvβ3 receptors and MDA-MB-231-fLuc human mammary cancer cells were purchased from the Academy of Military Medical Sciences (China) and cultured in Dulbecco’s modified Eagle’s medium (DMEM; Hyclone, China) supplemented with 10% FBS and 1% penicillin/streptomycin (Promega) at 37 °C in an atmosphere containing 5% CO_2_. Cells were seeded into 96-well microtiter plates at 10,000 cells per well. After incubation for 24 h, the cells were washed with phosphate-buffered saline (PBS), and the medium was replaced with fresh medium containing various concentrations of the MSNs or ICG/MSNs-RGD (30, 50, 100, 150, 200, 250, 300, 350, and 400 μg/mL). An equivalent volume of fresh medium (200 μL/well) was added to the control wells. After 24 h incubation, the treated cells were washed with PBS, and then fresh medium (100 μL) and MTT solution (10 μL, 5 mg/mL in PBS) were added to each well and cultured for another 4 h. The old medium was removed, and dimethyl sulfoxide (DMSO; 120 μL; China) was added to each well. The absorbance at 490 nm was read using a microplate reader to calculate relative cell viabilities.

### Integrin binding assays

To evaluate specificity for the integrin α_v_β_3_ receptor, we used MDA-MB-231-fLuc and MCF-7 (Department of Radiology, Peking Union Medical College Hospital, China) cells, which exhibited high and low integrin α_v_β_3_ expression, respectively. For this, 10^5^ cells were cultured in 96-well microtiter plates for 24 h prior to the addition of ICG, ICG/MSNs, or ICG/MSNs-RGD (the ICG concentration was 5 μg/mL) for 3 h. After washing three times with cell culture medium and twice with PBS, culture medium (100 μL) was added to each well, and the absorbance at 780 nm was measured with a microplate reader.

### *In vitro* cell fluorescence imaging

MDA-MB-231-fLuc and MCF-7 cells were incubated with ICG/MSNs-RGD (0.2 mg/mL) for 3 h. The cells were washed three times with cell culture medium and twice with PBS, and then additional cell culture medium (2 mL) was added for continuation of cell culture. A split fluorescence microscope and NIR-fluorescence imaging-guided surgery system were used to observe the cells at excitation and emission wavelengths of 780 and 840 nm, respectively.

### Mouse models and *in vivo* fluorescence imaging

All animals were purchased from the Department of Experimental Animals at Peking University Health Science Center (China). All experimental protocols were approved by the Institutional Animal Care and Ethics Committee of Zhujiang Hospital of Southern Medical University, and all the methods were carried out in accordance with the approved guidelines.

A subcutaneous hepatic tumour model was established by subcutaneous injection of 2 × 10^6^ Hep-G2-GFP-fLuc cells into the left upper elbow/back region in 6–7-week-old BALB/c *nu/nu* male mice.

An orthotopic hepatic cancer model was established by injecting 2 × 10^6^ Hep-G2-GFP-fLuc cells into the livers of 6–7-week-old male BALB/c *nu/nu* mice in a vehicle of Matrigel (4 mg/mL; BD Biosciences) in PBS with a 50-μL injection volume. Tumours were allowed to grow for 12 days, and then visualised with an IVIS Spectrum Imaging System (PerkinElmer, Germany). For imaging, mice were anesthetised with a 3% isoflurane (Beijing Yeeran Technology Limited, China)/air mixture, injected with luciferin solution (80 μL, 15 mg/mL; Fremont, CA, USA), and then imaged 8 min later in the supine position.

For the metastatic breast cancer model, 2 × 10^6^ MDA-MB-231-fLuc cells were injected into 6–7-week-old female BALB/c *nu/nu* mice in a vehicle of Matrigel (4 mg/mL) in PBS with a 50-μL injection volume. Tumours were allowed to grow for 12 days prior to monitoring with IVIS as described above.

Six mice with subcutaneous hepatic tumours measuring more than 5 mm in diameter were selected for *in vivo* fluorescence imaging, anesthetised with 3% isoflurane/air, and treated with ICG/MSNs or ICG/MSNs-RGD (150 μL, 0.2 mg/mL dispersed in PBS, n = 3/group) via tail vein injection. Mice were monitored by IVIS at 10 min, 24 h, 48 h, 72 h, 96 h, and 120 h postinjection at excitation and emission wavelengths of 780 and 840 nm, respectively. Imaging data were analysed with IVIS Living Image 3.0 software (PerkinElmer).

### Surgical residual verification

Surgical studies were performed by two general surgeons (Zhujiang Hospital, China). Twelve mice (six mice per surgeon) were treated with ICG/MSNs-RGD (150 μL, 0.2 mg/mL) 48 h prior to surgery. Surgeons were asked to remove tumour tissue with maximum protection of the normal tissue based on visual inspection and palpation. After this procedure, NIR and GFP images were acquired to determine whether residual tumour tissues were present. If tumour tissue remained, the surgeons continued the resection with imaging guidance until all tumour tissue was removed. Tumour numbers and sizes were recorded, and the resected tissue was histologically examined.

### Intraoperative detection of metastatic lesions

Through the continuous monitoring of 20 mice with orthotopically implanted liver tumours (Hep-G2-GFP-fLuc cells) using bioluminescence imaging, we found intrahepatic metastases in three mice and peritoneal satellite lesions in five other mice. Surgical studies were performed by a general surgeon on animals administered ICG/MSNs-RGD (150 μL, 0.2 mg/mL) 48 h prior to surgery. Both orthotopic and metastatic liver cancer model mice were anesthetised with 4% chloral hydrate (120 μL, 98%; Alfa Aesar) solution and fixed on the operational bed in the supine position. The abdominal cavities were exposed with a longitudinal incision in the middle of the abdomen. The surgeon then resected all the primary and metastatic tumour tissue under the guidance of the NIR-fluorescence imaging system. The TBR ratio for each ICG/MSNs-RGD probe was evaluated by drawing 10 regions of interest (ROIs; 0.2 mm^2^) within the tumour nodules and adjacent liver tissue and measuring their average fluorescence intensity. The TBR value was then calculated as the average fluorescence intensity of the tumour nodule divided by that of surrounding areas.

### Histological analysis and NIR imaging of resected specimens

Excised tissues were embedded in optimum cutting temperature (OCT) compound (Leica, Germany), snap-frozen in liquid nitrogen, and stored at –80 °C. Sections were cut on a cryostat microtome (Leica) at a thickness of 8 μm and stained with H&E. Following pathological examination, ICG/MSNs-RGD-based fluorescence images were collected using an NIR-fluorescence imaging system to determine the fluorescence signal differences between tumour tissues and normal tissues.

### Statistical analysis

Statistical analysis was performed using SPSS v.20 (IBM Software, USA). Data are expressed as the means ± standard deviations (SDs). Two-tailed, independent two-sample t-tests were used to assess differences in the optical densities and fluorescence intensities between groups. Differences with *p* values of less than 0.05 were considered significant.

## Additional Information

**How to cite this article**: Zeng, C. *et al*. Intraoperative Identification of Liver Cancer Microfoci Using a Targeted Near-Infrared Fluorescent Probe for Imaging-Guided Surgery. *Sci. Rep*. **6**, 21959; doi: 10.1038/srep21959 (2016).

## Supplementary Material

Supplementary Information
