# Supplementary material for: Intraoperative Identification of Liver Cancer Microfoci Using a Targeted Near-Infrared Fluorescent Probe for Imaging-Guided Surgery
Source: Sci Rep. 2016 Feb 29;6:21959. doi: 10.1038/srep21959 (PMC4770417; doi:10.1038/srep21959)
Supplement: Supplementary Information [file srep21959-s1.pdf]

Intraoperative Identification of Liver Cancer Microfoci Using a Targeted Near-Infrared Fluorescent Probe for Imaging-Guided Surgery

Chaoting Zeng[1,2†], Wenting Shang[2,3†], Kun Wang[2,3†], Chongwei Chi[2,3†], Xiaohua Jia[2,3], Cheng Fang[1], Yang Du[2,3], Jinzuo Ye[2], Chihua Fang[1], and Jie Tian[2,3*]*

[1]Department of Hepatobiliary Surgery, Zhujiang Hospital, Southern Medical University, Guangzhou 510280, China.
[2]Key Laboratory of Molecular Imaging, Institute of Automation, Chinese Academy of Sciences, Beijing 100190, China.
[3]Beijing Key Laboratory of Molecular Imaging, Beijing 100190, China.

[†]The parallel first authors
Mr. Chaoting Zeng, Dr. Wenting Shang, Prof. Kun Wang, and Dr. Chongwei Chi

[*]The parallel corresponding authors

Prof. Jie Tian, Ph.D., Fellow of IEEE, SPIE, IAMBE, AIMBE, IAPR
President of the Chinese Society for Molecular Imaging
Professor and Director of the Key Laboratory of Molecular Imaging, Institute of Automation, Chinese Academy of Sciences
Professor and Director of Beijing Key Laboratory of Molecular Imaging
Zhongguancun East Road #95, Haidian Dist.
Beijing 100190, P. R. China
Phone: +86 10 82618465; Fax: +86 10 62527995
Email: tian@ieee.org

Prof. Chihua Fang
Director of the Department of Hepatobiliary Surgery,
Zhujiang Hospital, Southern Medical University,
No. 253, Gongye Avenue,
Guangzhou 510280, P.R. China
Phone: +86 20 61643208
Email: fangch_dr@126.com

Synthesis and characterisation of ICG/MSNs-RGD

Supplemental Figure 1.

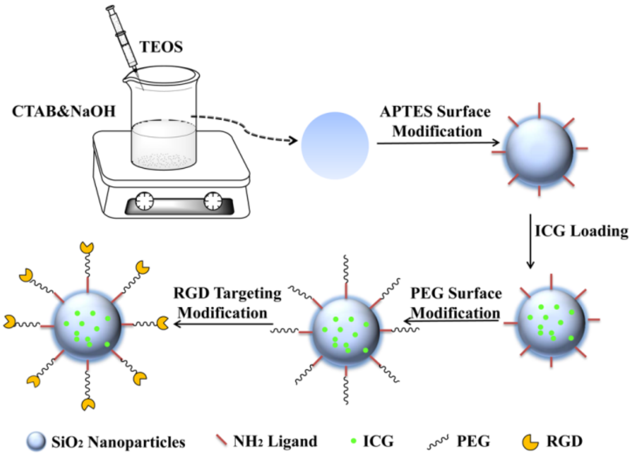

Supplemental Figure 1. Schematic of the synthesis of the ICG/MSNs and ICG/MSNs-RGD probe. The MSNs were developed using a hydrothermal process. The ICG/MSNs was prepared by loading ICG into the MSNs and coating with PEG; the subsequent product (ICG/MSNs-RGD) was generated by conjugating the ICG/MSNs with the RGD peptide.

Two fluorescent probes were synthesised for this study (Supplemental Fig. 1). ICG/MSNs, as a nontargeting probe, were prepared by loading ICG into the MSNs and coating with polyethylene glycol (PEG). The targeted probe ICG/MSNs-RGD was designed for tumour-selective delivery by conjugating ICG/MSNs with RGD, which selectively recognises the integrin $\alpha_v\beta_3$ receptor, which is overexpressed in many tumour types.

Supplemental Figure 2

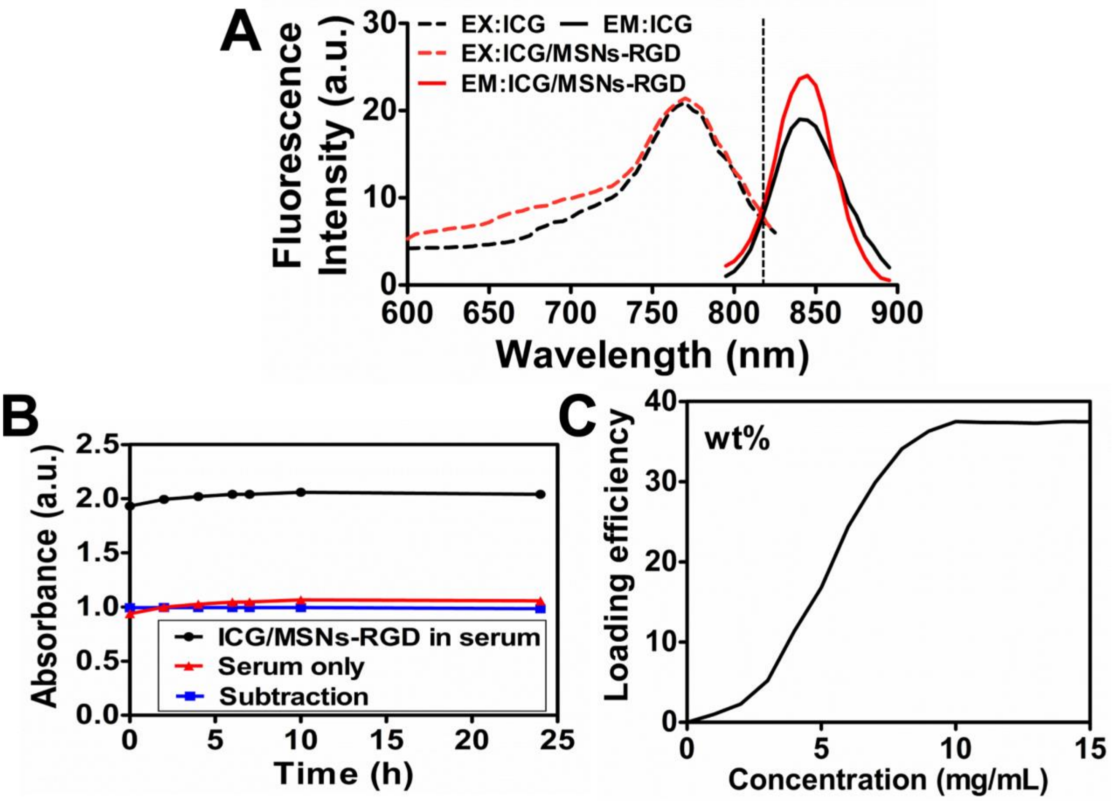

Supplemental Figure 2. (A) Fluorescence excitation and emission spectra of ICG and ICG/MSNs-RGD. (B) ICG/MSNs-RGD exhibited excellent optical stability in FBS. (C) ICG-loading efficiency of the MSNs reached a maximum (37.5%) when the ICG concentration was higher than 10 mg/mL. a.u.: arbitrary units. EX: excitation. EM: emission.

The excitation and emission spectra of ICG/MSNs-RGD and ICG were measured by fluorescence spectrofluorometry. The excitation spectrum of ICG/MSNs-RGD exhibited an absorption peak at 770 nm, and a fluorescence emission peak was observed at 837 nm. Both features were nearly the same as those observed for ICG (Supplemental Fig. 2A), indicating that the probe synthesis process did not change the optical properties of the ICG chromophore. Analysis of the absorbance stability of

ICG/MSNs-RGD in FBS revealed a slight increase over time, as measured by UV-Vis spectrophotometry (Supplemental Fig. 2B, black plots); however, after subtracting the background of FBS-only (Supplemental Fig. 2B, red plots), the difference between the two solutions appeared to be consistent at all time points (Supplemental Fig. 2B, blue plots), which is indicative of good absorbance stability and no aggregation of the probe. Moreover, the ICG maximal encapsulation efficiency in the MSNs was calculated to be 37.5% by UV-Vis spectrophotometry (Supplemental Fig. 2C).

In vitro evaluation of cytotoxicity and tumour cell specificity

Supplemental Figure 3

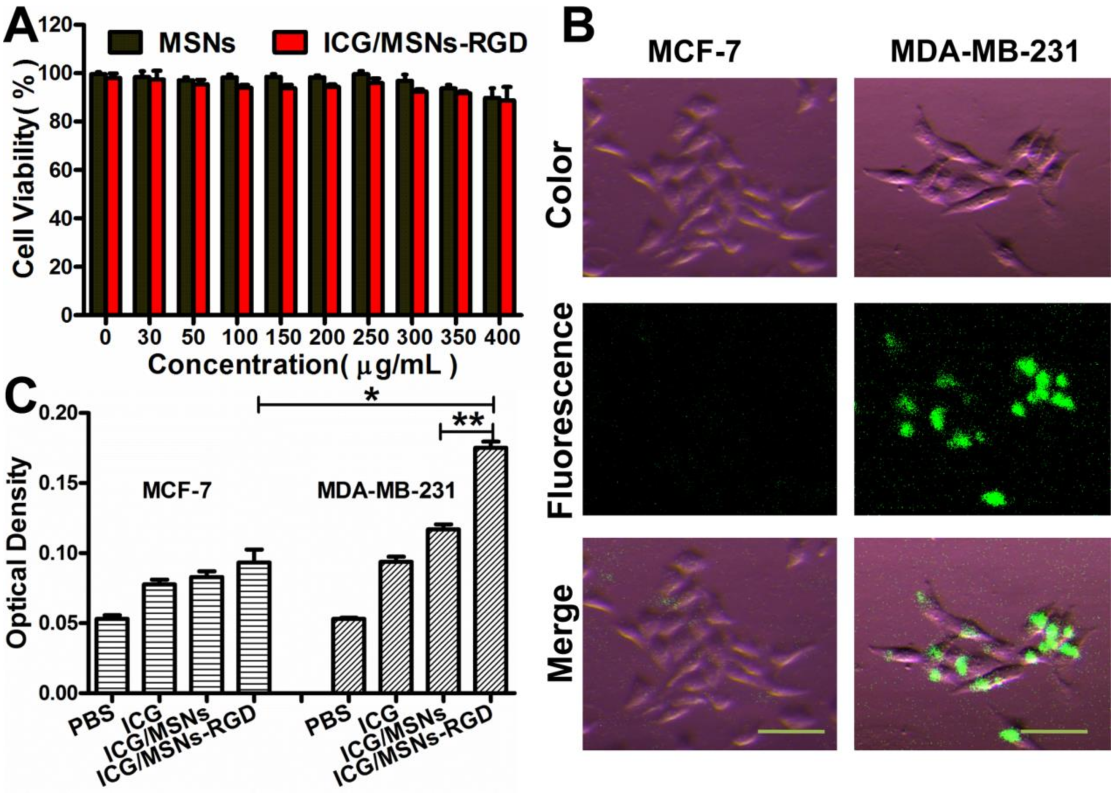

Supplemental Figure 3. Evaluation of the in vitro cytotoxicity and specificity of the nanoparticles. (A) Cell viability assays (Hep-G2-GFP-fLuc cells) were used to compare the toxicity of MSNs and ICG/MSNs-RGD at various concentrations. Experiments were run in triplicate. (B) Comparison of the in vitro uptake of ICG/MSNs-RGD between MDA-MB-231-fLuc cells (exhibiting high $\alpha_v\beta_3$ expression) and MCF-7 cells (exhibiting low $\alpha_v\beta_3$ expression), demonstrating high tumour cell specificity. Scale bar, 50 μm. (C) Comparison of the in vitro uptake of ICG, ICG/MSNs, and ICG/MSNs-RGD in MDA-MB-231-fLuc and MCF-7 cells. Experiments were run in triplicate. *$p < 0.05$, **$p < 0.01$.

MSNs and ICG/MSNs-RGD-mediated cytotoxicity was evaluated in green

fluorescent protein (GFP)-transfected luciferase-expressing human hepatocellular carcinoma (Hep-G2-GFP-fLuc) cells and MDA-MB-231-fLuc human breast cancer cells. Notably, the survival rate of Hep-G2-GFP-fLuc cells revealed that both types of nanoparticles exhibited excellent biocompatibility, as they had no significant effects on cell viability ($p > 0.05$) at various concentrations (Supplemental Fig. 3A). Similar results were also observed with MDA-MB-231-fLuc cells (Supplemental Fig. 5), further validating our hypothesis.

The differences in probe uptake between MDA-MB-231-fLuc and MCF-7 cells (two types of human breast cancer cells exhibiting high and low integrin $\alpha_v\beta_3$ expression, respectively) indicated satisfactory tumour specificity for the ICG/MSNs-RGD probe (Supplemental Fig. 3B). Furthermore, the quantitative analysis also confirmed this finding, a significantly higher uptake of ICG/MSN-RGD in MDA-MB-231 cells was verified by comparing with its uptake in MCF-7 cells ($P = 0.024 < 0.05$, Fig. 3C). The uptake of ICG/MSNs-RGD in MDA-MB-231-fLuc cells was significantly higher than that of the ICG/MSNs ($p = 0.001 < 0.01$), whereas no significant difference was found in MCF-7 cells ($p = 0.36 > 0.05$; Fig. 3C), suggesting that $\alpha_v\beta_3$-receptor-mediated binding may be the mechanism of uptake of the RGD-based probe.

Supplemental Figure 4

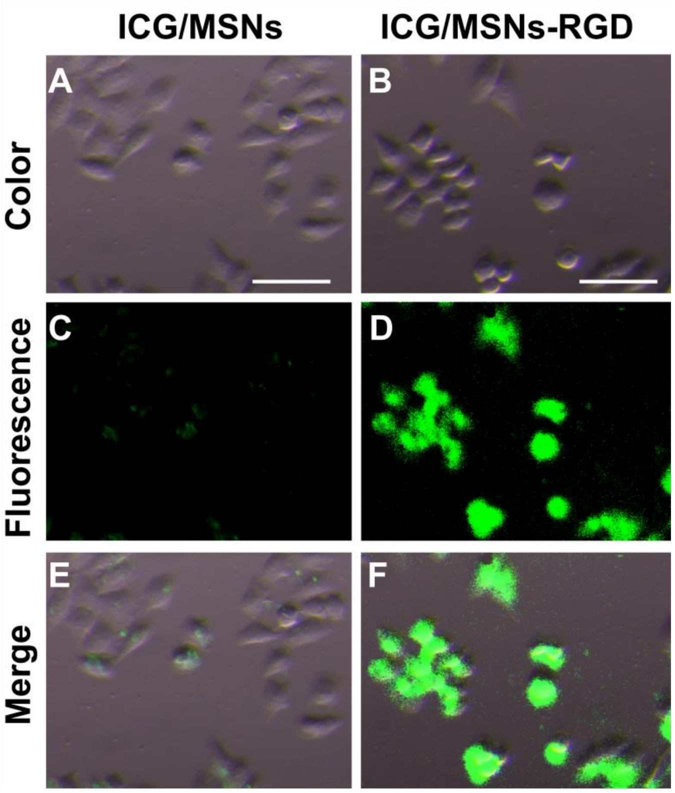

Supplemental Figure 4. The in vitro specificity of the nanoparticles to the Hep-G2-GFP-fLuc cells. The Hep-G2-GFP-fLuc cells were incubated with the active targeted probe (ICG/MSNs-RGD) and the untargeted probe (ICG/MSNs). The different fluorescent intensity between the ICG/MSNs-RGD group and the ICG/MSNs group suggests its good tumor specificity. Scale bar, 50 μm.

In order to further confirm the in vitro specificity of the ICG/MSNs-RGD probes to the Hep-G2-GFP-fLuc with high integrin $\alpha_v\beta_3$ receptor expression, the Hep-G2-GFP-fLuc cells were incubated with the active targeted probes (ICG/MSNs-RGD) (0.2 mg/mL) or the untargeted probes (ICG/MSNs) (0.2 mg/mL) for 3 h. They were washed three times with cell culture medium and twice with PBS, and then additional cell culture medium (2 mL) was added for continual cell culture. A split fluorescence microscope and NIR-fluorescence imaging-guided surgery

system were used to observe the cells at excitation and emission wavelengths of 780 and 840 nm, respectively. As is shown in Supplemental Fig. 4, compared with the the Hep-G2-GFP-fLuc cells incubated with the ICG/MSNs (Supplemental Fig. 4C and 4E), the cells incubated with the ICG/MSNs-RGD had brighter fluorescence (Supplemental Fig. 4D and 4F). This observation also makes us understand that the specific binding ability of the ICG/MSN-RGD probes to the Hep-G2-GFP-fLuc cells (with high integrin $\alpha_v\beta_3$ receptor expression) is well. And those data indicated that the uptake of the RGD-based probes is mostly mediated by integrin $\alpha_v\beta_3$ receptor affinity.

Supplemental Figure 5.

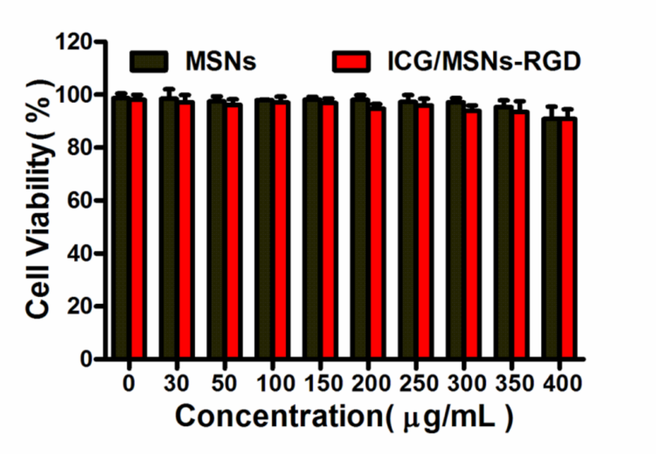

Supplemental Figure 5. Cell-viability assays (MDA-MB-231-fLuc cells) comparing the toxicities of MSNs and ICG/MSNs-RGD at various concentrations. No significant decrease of cell viability was found in those two groups. Experiments were run in triplicate.

Supplemental Figure 6

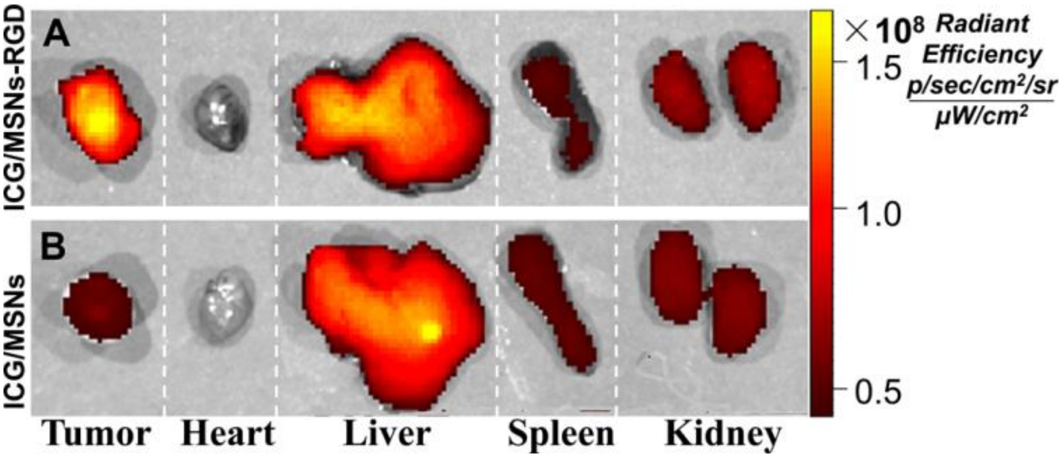

Supplemental Figure 6. Ex vivo fluorescence images of dissected organs from Hep-G2-GFP-fLuc xenografts. (A) The high fluorescence from the tumour suggested the remarkable tumour specificity of ICG/MSNs-RGD. (B) The low fluorescence from the tumour indicated reduced ICG/MSN uptake. The other organs showed similar fluorescence signals from the two probes. High fluorescence from the livers and low fluorescence from the kidneys in both cases revealed that the probes were excreted through the liver and digestive system.

Supplemental Figure 7

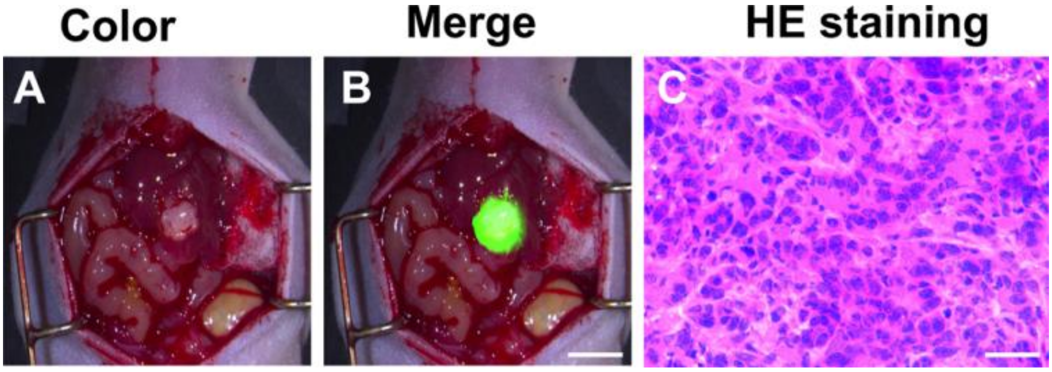

Supplemental Figure 7. Intraoperative identification of liver metastasis from breast cancer. (A, B) The ICG/MSNs-RGD-mediated NIR-FMI offered satisfactory delineation of tumour margins, despite the tumour being a transplanted breast tumour in the liver. Scale bar, 4 mm. (C) Histological analysis of the resected tissue specimen from (B) showed the typical architecture of MDA-MB-231-fLuc breast cancer. Scale bar, 100 μm.
